# Glycemic Control and Adherence to Treatment According to Personality, Self‐Esteem, and Perceived Stress in Type 1 Diabetes Mellitus

**DOI:** 10.1155/nrp/8711334

**Published:** 2026-04-15

**Authors:** Carmen Grau-Del Valle, Jose Francisco Marco-Expósito, Neus Bosch-Sierra, Juan Diego Salazar, Santiago García, Eva Solá, Inmaculada Montoya-Castilla, Carlos Morillas, Celia Bañuls

**Affiliations:** ^1^ Department of Endocrinology and Nutrition, University Hospital Doctor Peset-Foundation for the Promotion of Health and Biomedical Research in the Valencian Region (FISABIO), Valencia, Spain; ^2^ Department of Medicine, University of Valencia, Valencia, Spain, uv.es; ^3^ Department of Personality, Assessment and Psychological Treatment, University of Valencia, Valencia, Spain, uv.es

**Keywords:** adherence treatment, glycemic control, perceived stress, personality traits, self-esteem, Type 1 diabetes mellitus

## Abstract

The aim of this study was to assess whether personality traits, self‐esteem, and perceived stress are associated with glycemic control and adherence to treatment in adults with Type 1 diabetes mellitus (T1DM). We conducted a cross‐sectional study in 107 T1DM patients and measured psychological variables (self‐esteem, perceived stress, and personality traits) and clinical markers. More than 90% of patients had good or very good adherence to treatment. In addition, patients with poor adherence had higher HbA1c levels. Regarding personality traits, adherence to treatment correlated significantly with agreeableness and conscientiousness and negatively with neuroticism. Patients with high self‐esteem had greater adherence to treatment. Finally, a significant positive correlation was observed between perceived stress and HbA1c. We concluded that some personality traits and self‐esteem are associated with treatment adherence, and perceived stress is associated with poorer glycemic control in T1DM.

## 1. Introduction

Type 1 diabetes mellitus (T1DM) requires a multifactorial approach to achieve optimal health outcomes, integrating clinical care with behavioral strategies and attention to psychosocial aspects [1]. Beyond managing biomedical parameters, individuals living with T1DM must cope with substantial psychological and emotional demands that influence self‐management and adherence to treatment recommendations [2]. In adulthood, approximately 95% of diabetes management responsibilities fall on the individual, underscoring the importance of psychosocial resources for maintaining daily self‐care behaviors [3].

Among the psychosocial factors associated with T1DM outcomes, emotional distress, anxiety, depressive symptoms, personality traits, perceived stress and self‐esteem have all been linked to glycemic control, including glycosylated hemoglobin (HbA1c) levels [4–6]. Personality traits, relatively stable yet evolving dimensions encompassing behavioral, cognitive, and emotional patterns, play a significant role in shaping coping mechanisms in chronic illness [7, 8]. Traits such as conscientiousness, extraversion, and openness have been associated with better glycemic outcomes, whereas neuroticism has been linked to poorer control [9, 10]. Psychological stress also affects T1DM management, as higher perceived stress is associated with greater difficulty regulating blood glucose and higher HbA1c levels [11–14]. Evidence further suggests that stress may influence glycemic outcomes both directly and indirectly through its effect on treatment adherence [15]. Self‐esteem, understood as an individual’s subjective evaluation of self‐worth, has also been identified as an important determinant of diabetes self‐management. Adults with T1DM and lower self‐esteem experience more difficulty achieving glycemic control and may be more vulnerable to poor adherence and maladaptive coping behaviors [16–18].

Although these findings emphasize the relevance of psychosocial factors in adults with T1DM, the evidence base remains limited. Most research in this population relies on small samples or cross‐sectional designs, which restricts generalizability and the ability to draw stronger conclusions [7, 19, 20]. In contrast, studies in children and adolescents with T1DM are far more extensive, including larger cohorts, longitudinal designs, and systematic reviews that provide robust evidence regarding the influence of psychological variables on diabetes management [21, 22]. This discrepancy highlights a persistent gap in the literature.

Recent reviews reinforce that, compared with adolescent populations, adults with T1DM are comparatively understudied, particularly regarding the combined role of personality traits, perceived stress, and self‐esteem in relation to glycemic control and adherence to treatment. While previous studies have examined these factors individually, less is known about how they interact to influence diabetes management in adulthood.

To address this gap, the present study aims to assess whether personality traits, self‐esteem, and perceived stress are associated with glycemic control and adherence to treatment in adults with T1DM. By examining these psychological factors alongside key diabetes management outcomes, the study seeks to provide a more comprehensive understanding of how psychological and behavioral dimensions collectively shape disease management in this population.

## 2. Materials and Methods

### 2.1. Subjects

Between 2020 and 2022, a cross‐sectional study was conducted in 107 adults with T1DM who were consecutively recruited into the study at their regular medical visit to the endocrinologist and a diabetes educator from the Endocrinology and Nutrition Service of University Hospital Dr. Peset, Valencia, Spain. Participants did not receive financial compensation, and they all signed an informed consent form and were assured of data confidentiality.

The inclusion criteria required participants to be over 18 years old and to have a diagnosis of T1DM with at least 1 year of disease progression. Patients with type 2 diabetes mellitus (T2DM) and those with difficulties in understanding and adequately answering the questionnaires were excluded from the study (people who could neither read nor write). The study adhered to the Declaration of Helsinki guidelines for research involving human subjects, and the Ethics Committee of University Hospital Dr. Peset approved all procedures (CEIm:94/19).

### 2.2. Variables and Instruments

#### 2.2.1. Glycemic Control

To evaluate the glycemic control, plasma HbA1c was measured by reversed‐phase chromatography HPLC as a direct method and clinical indicator.

#### 2.2.2. Adherence to Treatment

To objectively assess adherence to treatment, individuals with T1DM completed a validated questionnaire [15]. The questionnaire was composed of 25 items and 5 behavioral dimensions (insulin administration and medical visits, involvement in treatment, psychological variables, physical exercise, and nutritional guidelines). The items were evaluated with 6 types of responses on the Likert scale (1 = never, 2 = almost never, 3 = rarely, 4 = frequently, 5 = almost always, and 6 = always) to categorize the responses in a graded manner and subsequently organize the individuals into groups. The total score of the questionnaire ranged from 25 to 150 points, from which five ranges of adherence were established: 25 to 50 points (very poor adherence to treatment); 51 to 75 points (poor adherence to treatment); 76 to 100 points (satisfactory adherence to treatment); 101 to 125 points (good adherence to treatment); and 126 to 150 points (very good adherence to treatment). The questionnaire has adequate content and construct validity and high reliability (Cronbach’s alpha *α* = 0.92) [15].

#### 2.2.3. Self‐Esteem

The Rosenberg Self‐Esteem Scale was used to measure a person’s satisfaction with himself/herself. It consisted of 10 statements that the person must rate on a four‐point scale, ranging from “strongly disagree” to “strongly agree,” and half of the negatively directed statements are assigned inverse scores. The total score ranged from 10 to 40, from which three subscales of self‐esteem were established: 30 to 40 points (high), 25 to 29 (medium), and < 25 points (low). This instrument has been adapted into Spanish. The internal consistency, test–retest correlation, and reliability of the scale were good [23].

#### 2.2.4. Perceived Stress

The Spanish version (2.0) of the Perceived Stress Scale (PSS) was used to evaluate stress [24]. This tool evaluates the level of perceived stress by the patient during the previous month. It consists of 14 items with a Likert scale response format (0 = never, 1 = almost never, 3 = often, and 4 = very often). To arrive at the final result of the questionnaire, the scores of the items are summed, taking into account that items 4, 5, 6, 7, 9, 10, and 13 are inverted (0 = 4, 1 = 3, 2 = 2, 3 = 1, and 4 = 0). The total score ranges from 0 to 40, with a higher score indicating higher perceived stress. The internal consistency, test–retest correlation, and reliability of the scale were good in the Spanish population [25].

#### 2.2.5. Personality Characteristics

The NEO‐FFI Scale developed by McCrae and Costa (2008) is a personality test that measures five personality traits: neuroticism, extraversion, openness to experience, agreeableness, and conscientiousness [18]. Agreeableness (A) shows the qualities of empathy, altruism, kindness, cooperation, and acquiescence. Conscientiousness (C) characterizes individual differences in being punctual, determined, systematic, respectful, and trustworthy. Extraversion (E) reveals individual differences in sociability, decisiveness, positive emotionality, and excitement seeking. Neuroticism (N) indicates the degree of anxiety, negative feelings, low self‐esteem, and emotional stability. Finally, openness to experience (O) reflects individual differences in innovation, curiosity, self‐determination, and social attitudes. It consists of 60 items that the person must rate on a five‐point Likert scale, ranging from “strongly disagree” to “strongly agree”. The total score varies for each of the five traits, with a higher score indicating a higher level of that personality trait. The tool showed good content and construct validity, as well as good internal consistency [25]. Raw scores for each dimension were converted into standardized T‐scores following the instrument’s scoring manual. Based on these values, participants were categorized into five levels: very low (≤ 34), low (35–44), medium (45–54), high (55–64), and very high (≥ 65). This classification allowed for the identification of distinct personality profiles across the sample.

### 2.3. Statistical Analysis

Statistical analyses were performed using the SPSS version 22 statistical software. The mean and standard deviation were used to express clinical and sociodemographic characteristics, while frequency statistics were used to express qualitative variables. To compare HbA1c levels across adherence groups while controlling for potential confounders, an ANCOVA model was performed, with HbA1c as the dependent variable, adherence category as the fixed factor, and age, BMI, sex, and duration of illness included as covariates.

Pearson’s correlation coefficient was used to examine the relationship between psychological variables (perceived stress, self‐esteem, and personality traits) and the results of glycemic control (adherence to treatment and HbA1c), estimating a significant value of *p* < 0.05. Additionally, a multiple linear regression analysis was performed in which treatment adherence was analyzed as a continuous variable, with the aim of identifying the psychological and clinical factors that best predict it. The model included personality traits, self‐esteem, perceived stress, age, sex, and diabetes duration as independent variables.

## 3. Results

The study included a total of 107 individuals with T1DM, of whom 53.3% were female, with a mean age of 41.0 ± 12.2 years. With respect to glycemic control, the mean HbA1c was 7.8 ± 1.2%, and more than 90% of patients had good or very good adherence to treatment, while 5.8% and 2.9% had fair or poor adherence, respectively (Table 1).

**TABLE 1:** Clinical characteristics of the study population.

	**Mean (SD)**

*N*	107
Sex (% male/female)	46.7/53.3
Age (years)	41.0 (12.2)
BMI (kg/m^2^)	25.5 (3.6)
Duration of T1DM (years)	21.6 (12.0)
HbA1c (%)	7.8 (1.2)
Treatment Adherence	(%)
Very poor	—
Poor	2.9
Satisfactory	5.8
Good	41.7
Very good	49.5

A significant negative correlation was observed between HbA1c levels and the total score obtained on the treatment adherence questionnaire (Figure 1(a)). Moreover, when adherence was categorized into groups, individuals with poor adherence exhibited higher HbA1c levels (*p* < 0.001) with a 95% CI in poor adherence: 3.8–17.4; satisfactory adherence: 7.9–10.8; Good adherence: 7.3–8.0; very good adherence: 7.3–7.7 (Figure 1(b)). In the same way, the mean of HbA1c levels showed significant differences between the adherence to treatment groups after adjustment for age, BMI, sex, and duration of illness covariates.

FIGURE 1Relationship between adherence to treatment and glycemic control in T1DM patients. (a) HbA1c and adherence to treatment total score. (b) HbA1c and ranges of adherence to treatment. Values with different superscript letters (a, b, c) were significantly different (*p* < 0.05) when the four groups were compared by ANCOVA followed by post hoc test. Hence, means with the same superscript are not significantly different from each other (*p* > 0.05), while means that have no superscript in common are significantly different from each other (*p* < 0.05).

**Figure:**
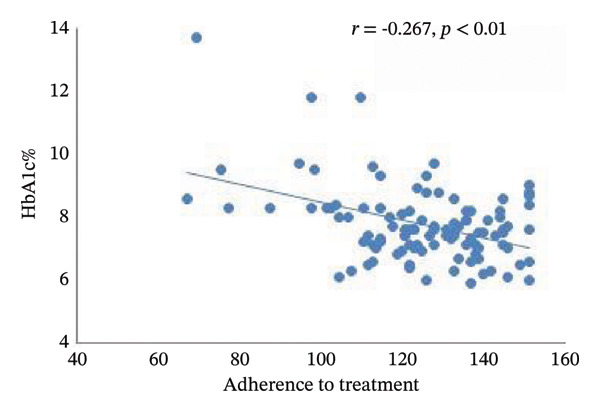
(a)

**Figure:**
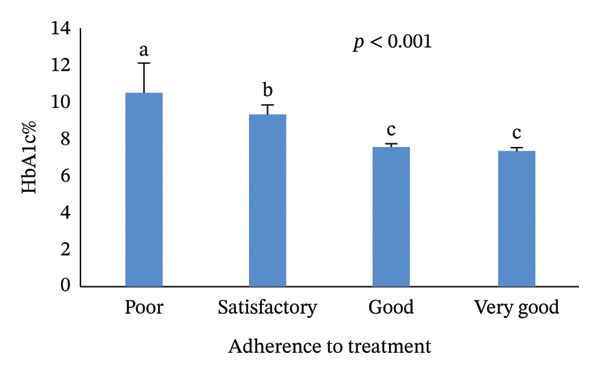
(b)

Regarding psychological variables, elevated levels of neuroticism were observed among personality traits, along with medium scores in openness to experience and conscientiousness and medium to high values in agreeableness and extraversion. High levels of self‐esteem were also predominant among participants. Most individuals demonstrated good or very good treatment adherence. The corresponding frequencies and distributions are presented in Table 2. The relationship between clinical variables and psychological aspects showed that adherence to treatment was positively correlated with agreeableness (*r* = 0.223; *p* < 0.024) and conscientiousness (*r* = 0.331; *p* < 0.001), and negatively correlated with neuroticism (*r* = −0.238; *p* < 0.016). The conscientiousness was also negatively and significantly associated with HbA1c (*r* = −0.209; *p* = 0.034). Individuals with high self‐esteem exhibited greater adherence to treatment (*r* = 0.220; *p* = 0.029), although self‐esteem was not significantly associated with HbA1c (*p* = 0.083). With respect to perceived stress, a statistically significant positive correlation was observed with HbA1c (*r* = 0.243; *p* < 0.05). In contrast, although it showed a trend, no statistical relationship was observed between perceived stress and adherence to treatment (*r* = −0.188; *p* = 0.062) (Table 3).

**TABLE 2:** Descriptive data for personality traits, self‐esteem, and perceived stress in the study population.

	**Very low**	**Low**	**Medium**	**High**	**Very high**

Personality Traits (%)					
Neuroticism (N)	1.9	12.1	26.2	35.5	24.3
Extraversion (E)	11.2	27.1	28	24.3	9.3
Openness to experience (O)	8.4	18.7	36.2	20.6	15.9
Agreeableness (A)	8.4	27.1	29.9	29.9	4.7
Conscientiousness (C)	19.6	22.4	32.7	18.7	6.5

	**Low**	**Medium**	**High**		

Self‐esteem (%)	5.6	11.1	82.8		
	Mean (SD)				
Perceived stress (0–56 points)	24.4 (9.5)				

**TABLE 3:** Correlation between personality traits, self‐esteem, perceived stress, adherence to treatment and HbA1c in the study population.

	**Adherence to treatment**	**HbA1c**

Neuroticism (N)	*r* = −0.238 *p* = 0.016	*r* = 0.052 n.s.

Extraversion (E)	*r* = 0.145 n.s.	*r* = −0.002 n.s.

Openness to experience (O)	*r* = −0.031 n.s.	*r* = −0.030 n.s.

Agreeableness (A)	*r* = 0.223 *p* = 0.024	*r* = 0.040 n.s.

Conscientiousness (C)	*r* = 0.331 *p* = 0.001	*r* = −0.209 *p* = 0.034

Perceived stress	*r* = −0.188 n.s.	*r* = 0.243 *p* < 0.05

Self‐esteem	*r* = 0.220 *p* = 0.029	*r* = −0.176 n.s.

Subsequently, a multiple linear regression was conducted to identify the most relevant predictors of treatment adherence. The model, with adherence as the dependent variable, showed an explanatory power of *R* = 0.225 and *R* ^2^ = 0.210, indicating that 21% of the variance in treatment adherence was explained by the model. Results revealed that both age (*β* = 0.285, *p* = 0.003) and conscientiousness (*β* = 0.306, *p* = 0.003) were significant predictors of adherence, suggesting that older individuals with higher levels of conscientiousness exhibited better treatment adherence.

## 4. Discussion

In relation to the aim of the study, the findings suggest that adherence to treatment is associated with personality traits such as agreeableness, conscientiousness, and neuroticism. Regarding HbA1c, conscientiousness was linked to this clinical variable. As for self‐esteem, individuals with T1DM who showed greater adherence to treatment also reported higher self‐esteem, whereas perceived stress was associated with HbA1c but not clearly with treatment adherence. Finally, perceived stress was related to higher neuroticism and lower conscientiousness.

Emotional aspects play a fundamental role in individuals with T1DM [26], as psychological and social problems can affect disease self‐management and consequently compromise health outcomes [27]. In line with the aim of our study, we found that stress is a common problem in this population, attributable to multiple stressors related to the disease itself [28]. Beyond its negative impact on general health, our findings support an association between perceived stress and elevated HbA1c levels, consistent with previous evidence indicating that psychological stress may negatively influence glycemic control [29]. Similar associations between stress and metabolic outcomes have been reported across developmental stages, suggesting that stress is a relevant factor throughout the lifespan in T1DM. However, in adults, stress may be more strongly linked to cumulative disease burden and daily life responsibilities. Although our cross‐sectional design does not allow establishing causal relationships, the observed associations underline the importance of addressing stress within T1DM management [13]. In our study, we did not observe a significant relationship between adherence to treatment and stress, although a trend was present. In contrast, other studies have reported associations between stress and adherence to treatment [30, 31]. This discrepancy may be related to the complex behavioral demands of diabetes self‐management (medication dosage, frequency and titration, glucose control, food intake, eating patterns, and physical activity) [32]. Thus, it is necessary to identify stressors that affect clinical management or glycemic outcomes through diabetes education and psychological interventions [33].

In this study, an association was also observed between poor adherence to treatment and higher HbA1c levels, although some investigations have not found a relationship between these two factors [3, 34]. This inconsistency may be explained by methodological differences, particularly in how adherence is measured. Self‐reported instruments assess individuals’ subjective perceptions, which may not fully reflect actual medication‐taking behavior [35]. Differences in measurement tools, potential reporting biases, and variability in study populations could therefore account for the divergent findings rather than the absence of a true association between adherence and HbA1c.

Regarding personality traits, we observed positive associations between adherence to treatment and characteristics such as conscientiousness and agreeableness, while a negative relationship was found with neuroticism. These findings align with previous research indicating that individuals with lower conscientiousness and higher neuroticism tend to show poorer adherence to medical recommendations and worse glycemic control [36, 37]. Much of this evidence, however, has been conducted in children and adolescents with T1DM. Our results suggest that these personality–adherence patterns extend into adulthood, highlighting a degree of continuity in psychological influences on diabetes self‐management across the lifespan [21, 22]. In addition, the multiple linear regression model indicated that both older age and higher levels of conscientiousness significantly predicted better treatment adherence. This suggests that, beyond personality traits, age‐related factors such as greater disease experience and self‐regulatory capacity may contribute to improved adherence behaviors in adults with T1DM. Nevertheless, the magnitude of these associations in adults may differ due to greater autonomy and longer disease experience. In our sample, the high levels of neuroticism observed may have amplified links between emotional reactivity, stress vulnerability, and difficulties in self‐management [38].

In addition to personality traits, we found that higher self‐esteem was associated with better treatment adherence, supporting evidence that self‐esteem can act as a protective factor in chronic illness [17, 39]. However, we did not find a direct association between self‐esteem and HbA1c levels. This suggests that, in adults, self‐esteem may influence metabolic outcomes more indirectly through behavioral pathways such as adherence or be moderated by factors like stress, mental health, or social support [40, 41]. Interestingly, studies in adolescents often report stronger direct links between self‐esteem and metabolic outcomes, whereas in our adult sample the association was clearer with treatment adherence than with HbA1c. This difference may reflect developmental shifts in the psychological determinants of diabetes management.

The relationship between diabetes and psychological well‐being is likely bidirectional: living with T1DM can negatively affect mental health, while pre‐existing psychological difficulties may hinder effective disease management [42]. Individuals with diabetes frequently report poorer overall health and greater psychological distress compared to those without the condition [42]. Therefore, integrating psychological care into routine diabetes management is essential [43]. Clinical guidelines, including those from the American Diabetes Association, emphasize that behavioral management and adequate psychological well‐being are critical components for achieving treatment goals [44]. Furthermore, psychosocial interventions targeting self‐esteem, stress, and coping strategies have been shown to improve both HbA1c levels and mental health outcomes in people with T1DM [43, 45]. Taken together, these findings highlight both the continuity of psychological influences on diabetes outcomes across the lifespan and the need for more adult‐specific research, as most existing evidence has focused on pediatric and adolescent populations. The present study has certain limitations that should be considered. Firstly, it should be noted that the results obtained through an indirect method (questionnaire) are subjective, and therefore, there is a possibility of overestimating compliance and only partially identifying noncompliance. Secondly, the adherence questionnaire used was originally developed and validated by the same research team. Although it was applied here to an independent sample, future studies conducted by external research groups are recommended to provide an independent validation and to further strengthen the generalizability and robustness of the instrument’s psychometric properties. Furthermore, recruiting participants during scheduled appointments with the endocrinologist could overestimate treatment adherence results. The study was conducted on T1DM individuals from a single country and center. Large‐scale studies employing these approaches are needed to support the present findings and clarify the underlying mechanisms linking psychological variables and glycemic outcomes.

The study has several strengths, such as a large sample size of T1DM individuals with a similar gender distribution. In addition, this study makes several novel contributions by focusing on the adult population with T1DM, whereas prior research has predominantly concentrated on children and adolescents. It employs a comprehensive psychological assessment using validated scales in the Spanish population to measure various psychological variables, providing a nuanced understanding of their impact on diabetes management. Notably, it identifies specific personality traits, such as agreeableness, conscientiousness, and neuroticism, that correlate with treatment adherence and glycemic control and highlights the significant impact of perceived stress on HbA1c levels in adults.

This study suggests that understanding the personality traits, self‐esteem levels, and perceived stress of individuals with T1DM may improve glycemic control through personalized treatment plans. Incorporating psychological assessments into routine diabetes care and implementing training program for healthcare providers can improve communication and adherence strategies. In addition, health policies should include psychological support services. For future research, we recommend conducting longitudinal and cross‐sectional studies, including diverse populations, and exploring additional psychological factors using multivariate analysis to identify significant predictors and useful categorizations. This research fills an important gap in the existing literature by focusing on adults with T1DM and using a comprehensive approach to assess psychological variables. These findings have practical implications for the development of targeted interventions aimed at improving the mental health and clinical outcomes of adults living with T1DM.

## 5. Conclusion

In conclusion, the study suggests that, on the one hand, in T1DM the personality traits (agreeableness, conscientiousness, and neuroticism) and self‐esteem are associated with adherence to treatment. On the other, perceived stress is associated with poorer glycemic control. Therefore, it is essential to identify and address the psychological factors that may be related to glycemic control in adults with T1DM to improve the management of the disease itself, generating changes in future intervention strategies and reducing potential long‐term complications. Thus, incorporating these aspects into educational and psychological programs for individuals with T1DM would be a key issue.

## 6. Relevance to Clinical Practice

Effective T1DM management requires a combined approach to boost treatment adherence and support self‐care. On one hand, diabetes education through nursing, and on the other, a psychological approach to support emotional well‐being, is essential. Together, they aim not only to improve medical and educational outcomes but also to ease the emotional stress tied to the daily demands of managing the disease.

## Author Contributions

Carmen Grau‐Del Valle, Jose Francisco Marco‐Expósito, Neus Bosch‐Sierra, Juan Diego Salazar, Santiago García, Eva Solá, Inmaculada Montoya‐Castilla, Carlos Morillas, and Celia Bañuls conducted the study. Carmen Grau‐Del Valle, Jose Francisco Marco‐Expósito, Neus Bosch‐Sierra, Santiago García, Eva Solá, Juan Diego Salazar, and Celia Bañuls provided overall supervision and the follow‐up of the volunteers in the study. Carmen Grau‐Del Valle, Inmaculada Montoya‐Castilla, and Celia Bañuls performed the data analyses and collected data. Carlos Morillas and Inmaculada Montoya‐Castilla assisted in the design and provided support throughout the course of the trial and analysis. Carmen Grau‐Del Valle, Eva Solá, Carlos Morillas, and Celia Bañuls performed statistical analyses, interpreted the data, and prepared the manuscript. Carlos Morillas and Celia Bañuls were responsible for its final content.

## Funding

This study was financed by grants PI18/00932, PI21/01160, and P24/01010 from the Carlos III Institute of Health and the European Regional Development Fund (ERDF, “A Way of Doing Europe”), and CIPROM/2022/32 from Generalitat Valenciana. C.G‐DV was a beneficiary of a PFIS contract (FI19/00076), and C.B. was a beneficiary of a Miguel Servet contract (CP19/00077) from the Carlos III Institute of Health. This study was cofunded by the European Commission, HORIZON EUROPE EU Programme (HORUS ‐Ref. 101136516) (CG‐dV).

## Disclosure

All authors read and approved the final version of the manuscript.

## Ethics Statement

The study was reviewed and approved by the appropriate ethics committees.

## Conflicts of Interest

The authors declare no conflicts of interest.

## Data Availability

The data that support the findings of this study are available on request from the corresponding author. The data are not publicly available due to privacy or ethical restrictions.
